# Editorial: Oxidative and Nitrosative Effects on the Biochemistry of Photosynthetic Algae and Plants Facing Global Change Conditions

**DOI:** 10.3389/fpls.2021.836752

**Published:** 2022-01-25

**Authors:** Susana Puntarulo, Andres A. Caro, Gabriela Malanga

**Affiliations:** ^1^Universidad de Buenos Aires, Facultad de Farmacia y Bioquímica, Fisicoquímica, Buenos Aires, Argentina; ^2^National Council for Science and Technology (CONICET)-Universidad de Buenos Aires, Instituto de Bioquímica y Medicina Molecular (IBIMOL), Buenos Aires, Argentina; ^3^Department of Chemistry, Hendrix College, Conway, AR, United States

**Keywords:** adaptation mechanisms, antioxidant capacity, global change, nitrogen radicals, oxygen radicals (ROS)

Although physiological and ecological effects of alterations in environmental conditions on photosynthetic algae and plants were postulated, the matter has not yet been fully elucidated. It has been suggested that instant responses of photosynthetic plants and algae to adverse environmental conditions involve excess production of reactive oxygen species (ROS) and reactive nitrogen species (RNS). Understanding the mechanisms by which photosynthetic algae and plants respond and adapt to environmental challenges is crucial to develop adaptive strategies for maintaining agricultural productivity and ecosystem services. For example, phytoplankton is the most significant primary producer in the ocean sustaining the pelagic food chains in the aquatic ecosystems, and is a substantial sink for CO_2_ in marine ecosystems. If these organisms are adversely affected, cell functionality, membrane stability, antioxidant enzyme activities, gene expression, and cell signaling in aquatic ecosystems would be put at risk from the lack of adequate food sources. This issue makes special focus on oxidative (i.e., related with ROS) and/or nitrosative (i.e., related with RNS) cellular balance on in photosynthetic plants and algae. This Research Topic focused on how oxidative and nitrosative metabolisms of photosynthetic organisms interact with climate change to affect their function. The four articles published here investigated different aspects of climate change effects.

The opinion article by Cabrera et al. reported the toxicological implications of the harmful marine phycotoxin domoic acid (DA) and its intrinsic properties. Special focus was made on the reported oxidative stress status in marine algae in relation to DA exposure. DA is released together with ROS into the cell culture medium. The short life span of ROS makes their effects not be very effective in damaging other organisms. DA release could allopathically affect other species of non-toxin-producing photosynthetic microalgae. Since Fe is known to be implicated in oxidative stress generation, the oxidative effects of DA were discussed in relation to its Fe-binding capacity. The effect of the DA-induced oxidative species burst on the antioxidant defense capacity was analyzed. Overall, the specific cellular response to the increased oxidative stress triggered by DA is suggested to be an important factor to allow survival of each organism, which contributes to determine how an increasing magnitude of the harmful algal bloom season produced by climate global change affects the composition of the community.

The opinion article by Del Castello et al. proposed that the expression of the chimeric globin-nitric oxide synthase from *Synechococcus* PCC 7335 (SyNOS) could improve cellular N metabolism enhancing N use efficiency (NUE) and crop production under N shortage conditions. The expression of the NOS from the algae *Ostreococcus tauri* (OtNOS) in tobacco increases growth rate, number of flowers and seed yield, but this phenotype is lost when plants are grown in low N condition. In contrast, SyNOS contains both the canonical oxygenase and reductase domains of NOS enzymes, and a globin domain suggested to be responsible for the oxidation of NO to ${\text{NO}}_{3}^{-}$. Engineered plants that express SyNOS, unlike the expression of other NOS, would remobilize N stored in Arg internal pools producing ${\text{NO}/\text{NO}}_{3}^{-}$ and may be an alternative strategy to favor crop productivity and stress tolerance. The hypothesis that the incorporation of Arg catabolic pathways in crops may improve N remobilization and plant productivity is further supported by the observation that the overexpression of arginases improves NUE and seed yield, in rice and cotton under different N conditions. Overall, the discovery of novel enzymes involved in NUE in photosynthetic organisms will help to generate crops to boost agriculture when confronting serious environmental challenges.

In the original research manuscript by Kennedy et al. the authors reported the use of microsensors to examine the influence of rapid shifts in irradiance on extracellular oxidative free radicals produced by sea-ice algae. Bottom-ice algal communities were exposed to one of three levels of incident light for 10 days. After 10 days, the snow cover was reversed, resulting in a rapid change in irradiance at the ice-water interface. In treatments acclimated to low light, the subsequent exposure to high irradiance resulted in an increase in the production of hydrogen peroxide and an increase in nitric oxide concentration after 24 h. In contrast, there was no significant response in sea-ice algae acclimated to high light and then exposed to a significantly lower irradiance at either 24 or 72 h. These results demonstrated that microsensors can be used to track real-time *in-situ* stress in sea-ice microbial communities.

In the original research manuscript by Essemine et al. the authors reported the contrasting responses of photosynthesis to salt stress in two C4 species: a glycophyte *Setaria viridis* (SV) and a halophyte *Spartina alterniflora* (SA). The effect of short-term salt stress treatment on the photosynthetic CO_2_ uptake and electron transport were investigated in SV and its salt-tolerant close relative SA. SV was incapable of surviving subjected to about 100 mM NaCl, and SA can tolerate concentrations up to 550 mM with slight effects on photosynthetic CO_2_ uptake rates and electron transport chain conductance. The results showed an enhancement in the P700 oxidation with increasing O_2_ concentration for SV following NaCl treatment and almost no change for SA. An activation of the cyclic NAD(P)H dehydrogenase-dependent pathway in SV was observed upon exposure to 50 mM NaCl; however, its activity in SA dropped as compared to the control. By using plastid terminal oxidase (PTOX) inhibitors the presence of a possible PTOX activity under salt stress for SA but not for SV was observed. In addition, an increase in PTOX amount for SA (but not SV) under salt stress was observed. Overall, this study provides strong proof for the existence of PTOX as an alternative electron pathway in C4 species (SA), which might play more than a photoprotective role under salt stress.

In closing, the articles in this Research Topic add to our knowledge of climate change and oxidative/nitrosative balances in photosynthetic algae and plants facing the global change conditions, while illustrating the need for more such research, such as the need for studies that examine interactive effects of multiple climate-change factors, and effects on interactions between plants and other organisms.

## Author Contributions

All authors listed have made a substantial, direct, and intellectual contribution to the work and approved it for publication.

## Funding

SP was supported by grants from the University of Buenos Aires (UBACyT 20020170100199BA) and National Council for Science and Technology (CONICET PIP 11220170100539CO). GM was supported by grants from National Council for Science and Technology (CONICET PIP 0635).

## Conflict of Interest

The authors declare that the research was conducted in the absence of any commercial or financial relationships that could be construed as a potential conflict of interest.

## Publisher's Note

All claims expressed in this article are solely those of the authors and do not necessarily represent those of their affiliated organizations, or those of the publisher, the editors and the reviewers. Any product that may be evaluated in this article, or claim that may be made by its manufacturer, is not guaranteed or endorsed by the publisher.

